# Effect of Water Ingress on the Mechanical and Chemical Properties of Polybutylene Terephthalate Reinforced with Glass Fibers

**DOI:** 10.3390/ma14051261

**Published:** 2021-03-07

**Authors:** Catarina S. P. Borges, Alireza Akhavan-Safar, Eduardo A. S. Marques, Ricardo J. C. Carbas, Christoph Ueffing, Philipp Weißgraeber, Lucas F. M. da Silva

**Affiliations:** 1 Instituto de Ciência e Inovação em Engenharia Mecânica e Engenharia Industrial (INEGI), 4200-465 Porto, Portugal; cspborges@fe.up.pt (C.S.P.B.); aakhavan-safar@inegi.up.pt (A.A.-S.); rcarbas@fe.up.pt (R.J.C.C.); lucas@fe.up.pt (L.F.M.d.S.); 2 Robert Bosch GmbH, Corporate Research and Advance Engineering, 71272 Renningen, Germany; Christoph.Ueffing@de.bosch.com (C.U.); Philipp.Weissgraeber@de.bosch.com (P.W.); 3 Departamento de Engenharia Mecânica, Faculdade de Engenharia (FEUP), Universidade do Porto, 4200-465 Porto, Portugal

**Keywords:** aging, moisture, diffusion, contamination, Fickian distribution, polybutylene terephthalate, short glass fiber, housing for electronic components

## Abstract

Short fiber reinforced polymers are widely used in the construction of electronic housings, where they are often exposed to harsh environmental conditions. The main purpose of this work is the in-depth study and characterization of the water uptake behavior of PBT-GF30 (polybutylene terephthalate with 30% of short glass fiber)as well as its consequent effect on the mechanical properties of the material. Further analysis was conducted to determine at which temperature range PBT-GF30 starts experiencing chemical changes. The influence of testing procedures and conditions on the evaluation of these effects was analyzed, also drawing comparisons with previous studies. The water absorption behavior was studied through gravimetric tests at 35, 70, and 130 °C. Fiber-free PBT was also studied at 35 °C for comparison purposes. The effect of water and temperature on the mechanical properties was analyzed through bulk tensile tests. The material was tested for the three temperatures in the as-supplied state (without drying or aging). Afterwards, PBT-GF30 was tested at room temperature following water immersion at the three temperatures. Chemical changes in the material were also analyzed through Fourier-transform infrared spectroscopy (FTIR). It was concluded that the water diffusion behavior is Fickian and that PBT absorbs more water than PBT-GF30 but at a slightly higher rate. However, temperature was found to have a more significant influence on the rate of water diffusion of PBT-GF30 than fiber content did. Temperature has a significant influence on the mechanical properties of the material. Humidity contributes to a slight drop in stiffness and strength, not showing a clear dependence on water uptake. This decrease in mechanical properties occurs due to the relaxation of the polymeric chain promoted by water ingress. Between 80 and 85 °C, after water immersion, the FTIR profile of the material changes, which suggests chemical changes in the PBT. The water absorption was simulated through heat transfer analogy with good results. From the developed numerical simulation, the minimum plate size to maintain the water ingress unidirectional was 30 mm, which was validated experimentally.

## 1. Introduction

Composite materials, and especially fiber-reinforced polymers (FRP), have been increasingly representing a low-weight alternative to conventional materials such as steel, as they present high specific properties such as stiffness and strength [1,2]. Additionally, they contribute to the reduction of the cost of the manufacturing process. FRPs can use both long and short fiber inclusions. Short fiber reinforced polymers (SFRP) are more suitable for manufacturing processes such as injection molding and promote an improvement of the mechanical properties than the fiber-free polymer.

One of the challenges related to the use of multiple polymeric materials, and their composites, is their sensibility to environmental conditions, such as corrosive environments, high temperatures, or humidity [3]. FRP can even be subjected to severe environmental conditions when they are used in infrastructures immersed in water such as fixed or floating composite bumper systems, for instance in bridges or in structures to support solar panels in water [4]. For these applications, new materials have been developed, such as cellulose nanocrystals cement paste [5], which can significantly improve the fracture behavior of the cement as well as retard the hydration of the cement, making it more suitable for these severe applications. However, since these materials are still under development, further research is needed. Moisture generally promotes degradation of the polymer, which can happen even when subjected to usual atmospheric humidity [6,7]; therefore, it is crucial to understand how the properties of composite materials change when exposed to water.

Water can be absorbed by the polymer as free and bound water. Free water occupies the free spaces on the structure of the material, the polymeric chain, which means it does not change the volume of the material. Bound water can increase the volume of the material, as it forms chemical bonds with the polymer’s molecular chain [3,6,8,9]. The bound water is also responsible for the decrease in glass transition temperature, $T_{g}$. Free water can start forming hydrogen bonds with the structure of the material, turning into bound water, a change that is promoted by high temperatures and longer exposure times [10,11].

Water diffusion is commonly described with good approximation by Fick’s law [12,13,14,15]. However, materials can exhibit Fickian or non-Fickian behavior, typically depending on the relation between the diffusion and relaxation rate. If the diffusion rate is much quicker than the relaxation rate, the material tends to exhibit Fickian behavior; however, if the relaxation rate influences the water uptake, non-Fickian behavior is usually identified [16,17,18]. In composite materials, it is often considered that the glass fibers are hydrophobic, absorbing significantly less water than the polymeric matrix, which is considered hydrophilic. However, they present preferential paths for the water ingress, and the interface between fiber and matrix is usually degraded. Therefore, degradation of thermoplastic composites is highly dependent on the specific composition of the matrix, the type of fiber, the fiber/matrix interface, and the volume fraction of fibers [13].

Regarding the mechanical properties, moisture degradation is generally responsible for a decrease in the strength and stiffness of thermoplastic materials. This process also contributes to a higher ductility of the polymer, verified by an increase in strain at failure, especially for temperatures above $T_{g}$ when the motion of the polymer’s molecular chain increases [13,18,19].

PBT (polybutylene terephthalate) is a particularly interesting polyester due to its rapid crystallization, good processing characteristics for short extrusion and injection molding, excellent mold flow, and improved flexibility, since it has two additional methylene units in the main chain when compared to polyethylene terephthalate (PET) [17,20,21]. However, PBT has limited toughness at low temperatures and high deformation rates. Additionally, with hygrothermal aging, it tends to undergo hydrolysis and become more brittle [22]. Hygrothermal aging is the process in which the deterioration of a given material occurs due to the action of moisture and temperature, as discussed above [21].

PBT reinforced with 30% short glass fiber, PBT-GF30, is widely used in the housing for electronic components due to its good properties and suitability for manufacture through injection molding. Its fast crystallization rate enhances moldability and, consequently, leads to parts with good surface quality [23,24].

However, while this material has many manufacturing advantages, it was identified that there are few recycling options. Research is being carried out in this field, most studies being performed fairly recently, since this is a material that suffered a great expansion in the past years due to its applicability in the automotive and electric sectors, among others [25]. The processes discussed are mainly focused on the depolymerization of PBT in supercritical methanol [26]. This research is important because when designing a new product, it is important not only take into consideration the long -term properties and durability of a component, but also be environmentally responsible for ensuring sustainable and responsible economic development, which not only benefits the environment but also the longevity and efficiency of the product [27,28,29].

In the housing for electronic components, PBT-GF30 can be subjected to humid environments at different temperatures. Therefore, it is important to know its water uptake behavior to predict the conditions at which the electronic components can be at risk and in contact with humidity. Additionally, this knowledge allows a more accurate design of the components for each specific application prior to its manufacturing, which reduces manufacturing costs of the component. As it has been said, one of the main issued regarding PBT usage is the recycling of the product; therefore, the low production cost of the components can be combined with a high waste management cost, which must be taken into account.

The moisture uptake of PBT and its glass fiber composites, with different volume fractions of fibers, was shown to follow the Fick’s law for water absorption regardless of the temperature of the water where the material is immersed [30]. Ishak et al. [21] also concluded that for short glass fibers, the desorption behavior is Fickian. In the same study, it was observed that the coefficient of diffusion decreased with increasing volume fraction of fibers. This occurrence was connected to the random orientation of the fibers, which retards the water ingress in the hydrophilic matrix. The equilibrium moisture increases with decreasing fiber content, which was attributed to increase in volume fraction of the hygroscopic PBT matrix. For fiber-free PBT and PBT with 18% short glass fiber, the coefficient of diffusion was determined. The coefficient of diffusion increased with increasing temperature, following an Arrhenius relationship.

Ishak et al. [21] also studied the effect of moisture aging on fiber-free PBT and PBT with 10% short glass fiber in volume at two different levels of relative humidity, 81.2 and 100%. Water has a significantly higher influence when the samples were subjected to a relative humidity of 100%. However, there are additional conclusions that can be drawn across all results. First, all the composites fractured in a brittle way, and a decrease in strength after aging was observed, which can be related to the yielding effect of absorbed water. For reinforced PBT, it was stated that failure occurs also due to the degradation of the interface between the fibers and the matrix. However, the glass fibers contributed to an improvement of the retention and recoverability of the tensile properties. With scanning electron microscopy (SEM) analysis, Ishak et al. [31] also observed hydrolysis of the matrix through the appearance of micro-voids and the absence of plastic deformation and degradation at the fiber–matrix interface. Additionally, regarding the variation of the mechanical properties of fiber-free PBT and PBT with 30% short glass fiber specimens saturated in humidity testing, after aging at 30 °C, both PBT and PBT-GF30 exhibited a slight decrease in strength compared to the unaged state. However, after aging at 90 °C, only 21% of the initial strength remained for fiber-free PBT and 36% for PBT-GF30.

Ishak et al. [32] studied the behavior of short fiber reinforced PBT immersed in water at 30, 60 and 90 °C for fiber contents between 0 and 18% in volume. For high temperature (90 °C), the toughness of the material decreases significantly. At this higher temperature, fiber-free PBT and PBT with 18% short glass fiber were analyzed through the determination of the pH and FTIR profile of the water where it has been immersed. Regarding the measurement of the pH, it was determined that it decreased after the immersion of PBT composites. Additionally, it has been observed that the difference between the initial pH and pH after 750 h of water immersion was higher for lower fiber contents. However, until slightly above 100 h of immersion, the pH decreased for both cases to the same value. Afterwards, the pH of the water where PBT with GF was immersed increased again due to the appearance of OH${}^{-}$ ions in the water. These results were confirmed through FTIR analysis of the water. The water where PBT was immersed revealed the presence of carboxylic groups associated with the hydrolysis of PBT. In the water where PBT-GF30 was immersed, there were also groups associated with the hydrolysis of glass fiber. These results indicate a hydrolysis of the fiber at high temperatures for long immersion times, which had been also detected by Hsu et al. [33].

Bergeret et al. [34] studied the effect of moisture on the properties of PET, PBT and PA6,6 reinforced with short glass fibers with different surface treatments. Regarding the PBT, when aged, the lack of chemical linkages between the glass fibers and PBT matrix creates a weak fiber/matrix interface and consequently, a fragile composite in a hygrothermal environment. Rosato et al. [23] also notes that although after 24 h fiber-free PBT only absorbs 0.1% of water, this material is not suitable for hot–humid environments at temperatures above 52 °C. Gardner et al. [35] studied the long-term resistance of fiber-free PBT, aging the material up to three years at relative humidities between 11 and 100% and temperatures between 66 and 93 °C and concluded that, above 50 °C, PBT loses half of its tensile strength in three to four years.

The main purpose of this work was the complete characterization of the water uptake behavior of PBT-GF30 and its effect on the properties of the material, while also considering the effect of temperature. It was also expected to understand how the experimental methods adopted to perform this characterization influence the results, comparing them with other studies. Numerically, it was intended to create a simple model to simulate the water uptake behavior of PBT-GF30. With the simulation established, the minimum plate length–thickness ratio for the water flow to be one dimensional was determined. Additionally, the mechanical performance of the bulk tensile tests was simulated to complement the experimental results.

## 2. Experimental Details

Housings for electronic components, composed of PBT-GF30, can be subjected to elevated temperatures between room temperature (RT) and temperatures of 130 °C during the manufacturing, packaging, transport, and storage processes. In this work, three different temperatures were analyzed, from a temperature slightly above RT, 35 °C, to the maximum temperature reported in the manufacturing process, 130 °C. The intermediate temperature was chosen to be 70 °C since it is also an interesting reference temperature of the process, being a relevant temperature for transport and packaging.

### 2.1. Materials

In this work, the PBT-GF30 short fiber, a polymeric matrix composite, was analyzed. To understand the effect of fiber content on the water uptake behavior of the material, fiber-free PBT was also used in this work. The material for the injection molded plates and respective data sheets were obtained from the manufacturer.

### 2.2. Specimen Manufacturing

For each test performed, suitable specimens were machined from plates of PBT-GF30 and PBT, previously obtained from injection molding. $T_{g}$ measurement specimens and standard “dogbone” specimens were obtained from 2 mm thick plates, and the gravimetric test specimens, differential scanning calorimetry (DSC) specimens, reduced-scale “dogbone” specimens, and FTIR specimens were obtained from 1 mm thick plates.

#### 2.2.1. Glass Transition Temperature

The water uptake behavior of the polymer is known to change if the temperature under analysis is above or below $T_{g}$. Therefore, the first step was to determine $T_{g}$. The determination of the glass transition temperature was carried out using rectangular specimens with 10 × 25 mm${}^{2}$ and 2 mm thickness. A hole with a 3.5 mm diameter was made in the center on the specimen to attach it to the apparatus used for $T_{g}$ measurement.

#### 2.2.2. Differential Scanning Calorimetry (DSC) and Fourier Transform Infrared Spectroscopy (FTIR)

For DSC and FTIR analysis, tests were performed using 1 mm thick disks with a 6 mm diameter. These specimens are typically used for DSC. However, due to their small size, they were also used for FTIR to minimize the time required to saturate the sample.

#### 2.2.3. Gravimetric Analysis

Plates used for the gravimetric study were manufactured based on the ISO 294-3 standard [36]. Therefore, plates of 60 × 60 mm${}^{2}$ and 1 mm thick were used.

The thickness is significantly smaller than all other dimensions so that it represents the preferential water absorption path and the water flow can be considered as one dimensional. However, these specimens require quite a large amount of material. Therefore, in Section 4, a study is conducted to attempt to obtain similar results with smaller plates.

#### 2.2.4. Bulk Tensile Tests

Standard “dogbone” bulk tensile specimens have large dimensions, and to study the effect of water on the mechanical properties of PBT-GF30, they should be immersed in water, which would require large water containers and long immersion times. Therefore, reduced-scale specimens were used to accelerate this process.

**Figure 1:**
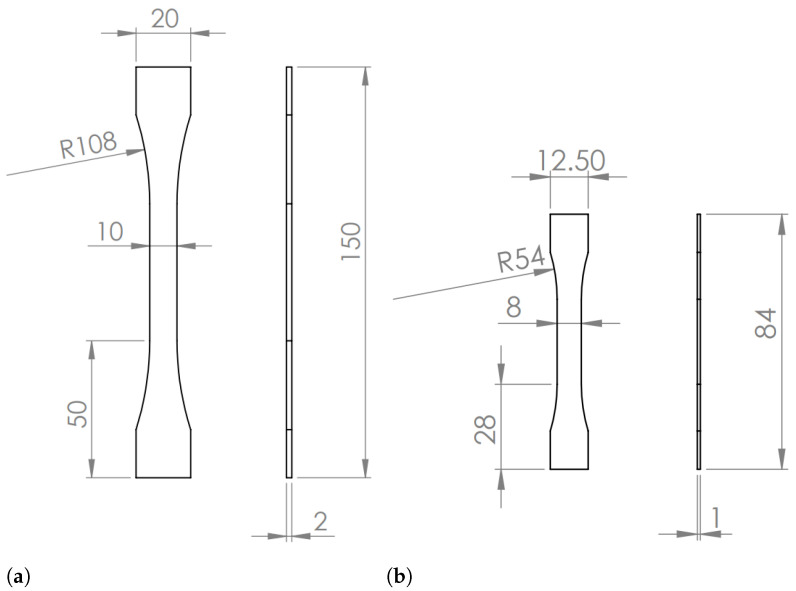
“Dogbone” specimens used for bulk tensile tests (dimensions in mm). (**a**) Standard specimens. (**b**) Reduced-scale specimens.

Standard “dogbone” specimens follow the British standard BS 2782 [37] (Figure 1a). The reduced-scale “dogbone” specimens have the dimensions shown in Figure 1b and have been used in previous studies [38,39].

### 2.3. Experimental Procedures

#### 2.3.1. Glass Transition Temperature

$T_{g}$ was measured using an in-house developed apparatus based on the DMA dynamic mechanical analysis concept [40]. The apparatus works by the measurement of the damping of a sample, which is kept vibrating at the resonance frequency as the temperature is increased. Temperature is measured using a dummy specimen, which is placed in the same oven of the specimen for damping measurement. As the specimen approaches $T_{g}$, the damping of the sample rises significantly, and the amplitude of the vibration approaches zero. As $T_{g}$ is surpassed, the amplitude increases again. Therefore, $T_{g}$ can be determined through the minimum of amplitude, or maximum of damping. For each condition, at least three specimens were tested.

#### 2.3.2. Differential Scanning Calorimetry (DSC)

The analysis of the DSC spectra of PBT and PBT-GF 30 was used to study the thermal behavior of the materials. These tests were carried out using a NETZSCH DSC 214 Polyma DSC21400A-0655-L machine. In the DSC test, the temperature changed between 20 and 300 °C; then, the samples were cooled from 300 to −20 °C and finally heated from −20 to 300 °C, which was the range considered for the analysis. The heating and cooling were performed at 10 °C/min in a N${}_{2}$ atmosphere. Three specimens were tested.

#### 2.3.3. Gravimetric Analysis

Before the immersion in water, the PBT and PBT-GF30 specimens were fully dried in silica for two weeks at 35 °C in order to ensure no water was found on the material. After the specimens were dried, their surface was manually treated with sandpaper (grade 800) to remove any contaminants on the surface and minimize surface roughness. The initial mass of the specimen was measured. Afterwards, the polymeric and composite plates were immersed in water, ensuring the plates were completely surrounded by water and not in direct contact with the container or other specimens so that the area of water absorption was not compromised. The temperature of water immersion was controlled by placing the containers with water in a cimatica chamber (Memmert GmbH, Büchenbach, Germany).

The mass of the plates was periodically measured until saturation was achieved. All mass measurements were performed using a microbalance with 0.1 mg of accuracy (KernToledo, Balingen, Germany). Before weighing the aged samples, their surface was cleaned using a paper towel. The water, or moisture, uptake of each specimen, for each time step is given by

(1) $$M_{t}=\frac{m_{t}-m_{0}}{m_{0}}\times 100\;\left(\%\right)$$

where $m_{t}$ is the mass of the specimen at the current time step and $m_{0}$ is the initial mass of the specimen. This process was repeated for 35, 70, and 130 °C. For 130 °C a water container was kept open inside the climatic chamber, and small amounts of water were periodically added. The specimens were considered to be saturated when the water content was stable, showing that the material was unable to absorb more water. For each condition, at least four specimens were tested.

#### 2.3.4. Bulk Tensile Tests

For the material in the as-supplied state, standard “dogbone” specimens were tested at room temperature, and reduced-scale “dogbone” specimens were tested at room temperature and at 35, 70, and 130 °C and after aging at the three temperatures.

The tensile tests were performed at the rate of 1 mm/min using an Instron^®^ 3367 universal testing machine with a load cell capacity of 30 kN.

For the tests at controlled temperature, the specimens were inserted in a climatic chamber, which was integrated in the universal testing machine. Prior to testing, each specimen was placed in the climatic chamber for a set period of time (10 min for the lower temperatures and 20 min for 130 °C) to ensure a uniform temperature distribution.

For the tests with water uptake for the three temperatures, the specimens were dried, as described for the gravimetric test specimens, and treated with sandpaper to create a uniformly rough surface; their mass was measured as mentioned above; and, finally, they were immersed in water. The specimens were tested periodically through the time needed to saturate the samples. Whenever the mechanical properties were measured, after a given immersion time, the surfaces of the specimens were dried with a paper towel, the mass was measured, and the tensile test was performed at RT.

For each condition, at least three specimens were tested.

#### 2.3.5. Fourier Transform Infrared Spectroscopy (FTIR)

The analysis of the FTIR spectra of dry samples and samples saturated at 35, 70, and 130 °C allows one to determine the extension of the effects of moisture aging, since if chemical relations are established between the PBT-GF30 and irreversible damage that occurs in the material, the intensity of the absorption bands for relevant groups changes. The specimens were simulated numerically and kept in water for a sufficient amount of time to reach saturation. The FTIR analysis was carried out using a PerkinElmer Spectrum Two machine (Waltham, MA, USA). Wave lengths of 4000–500 cm${}^{-1}$ were used at a scanning velocity of 0.2 cm${}^{-1}$. These tests were performed with a LiTaO${}_{3}$ detector (15,700–370 cm${}^{-1}$) and a KBr window. The spectra were obtained in attenuated total reflectance (ATR), which creates spectra equivalent to transmittance. Bulk specimens with a thickness of 1 mm were used. To narrow the range of temperatures at which changes in the FTIR profile occur in the material, other temperatures were, afterwards, analyzed. Three specimens were tested.

## 3. Experimental Results

### 3.1. Glass Transition Temperature

**Figure 2:**
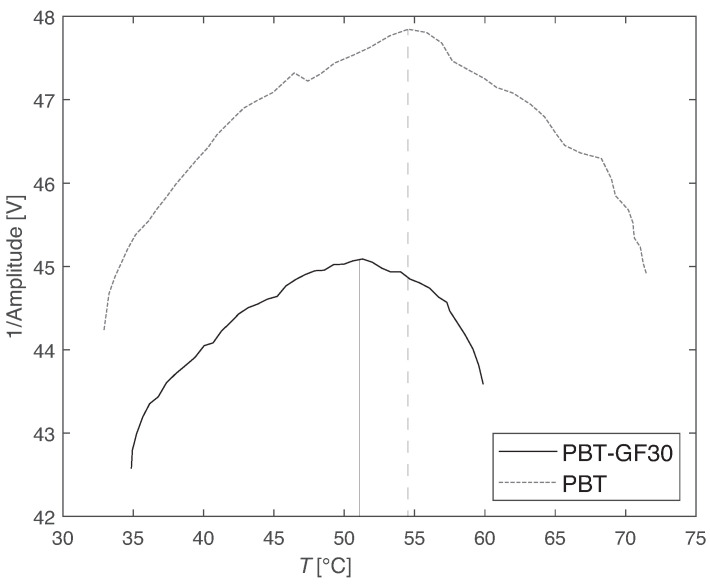
Glass transition temperature measurement results: representative curves.

Representative curves obtained from the $T_{g}$ measurement are shown in Figure 2, and the values of the $T_{g}$ are presented in Table 1.

The three levels of temperature analyzed are 15 °C below $T_{g}$ (35 °C), 20 °C above $T_{g}$ (70 °C), and 80 °C above $T_{g}$ (130 °C). These results are in accordance with the values reported by different authors, falling in the expected range between 40 and 60 °C [41,42,43,44].

### 3.2. Differential Scanning Calorimetry (DSC)

**Figure 3:**
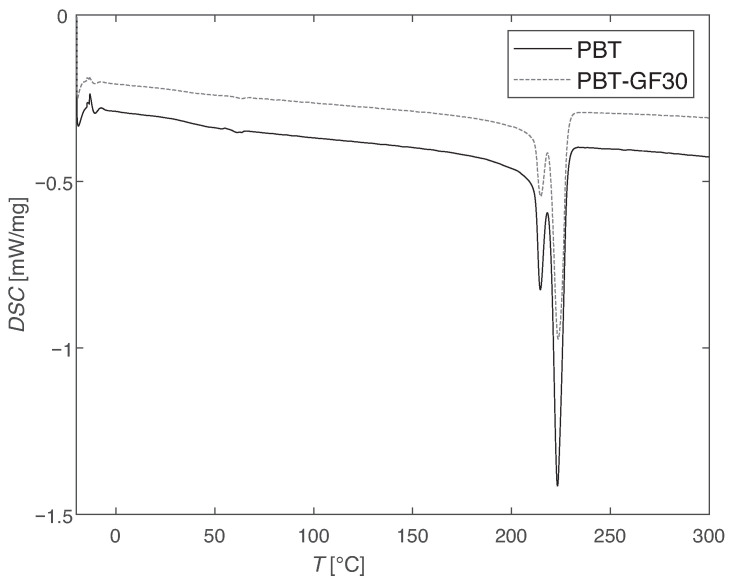
Differential scanning calorimetry (DSC) results: representative curves, where a peak is shown at around 220 °C.

The results from DSC analysis are presented in Figure 3.

It can be observed that both the PBT and the PBT-GF30 display a disturbance in the curve slightly above 50 °C, which corresponds to $T_{g}$, and only display significant degradation for temperatures above 200 °C, with a peak slightly above 220 °C, which corresponds to the melting temperature of the material. Therefore, in the range of temperatures analyzed, the chemical degradation is not significant. Similar results for DSC were found by Ma et al. [45] and Souilem et al. [46] for fiber-free PBT.

### 3.3. Gravimetric Analysis

A Fick’s law of diffusion can be fitted to the experimental results for the four conditions studied. The Fick’s law is given by [47]

(2) $$M_{t}=\left[1-\frac{8}{\pi^{2}}\overset{\infty }{\underset{n=0}{\sum }}\frac{1}{{\left(2n+1\right)}^{2}}\exp\left(\frac{-D{\left(2n+1\right)}^{2}\pi^{2}t}{4h^{2}}\right)\right]M_{\infty }$$

where *t* represents time starting from the immersion and *h* represents the thickness of the specimen. Therefore, a script was run in Matlab to determine the best fit of the coefficient of diffusion, *D*, and infinite water uptake, $M_{\infty }$. For that, the least square method was used. The values determined for each material and each temperature, *T*, are given in Table 2.

**Figure 4:**
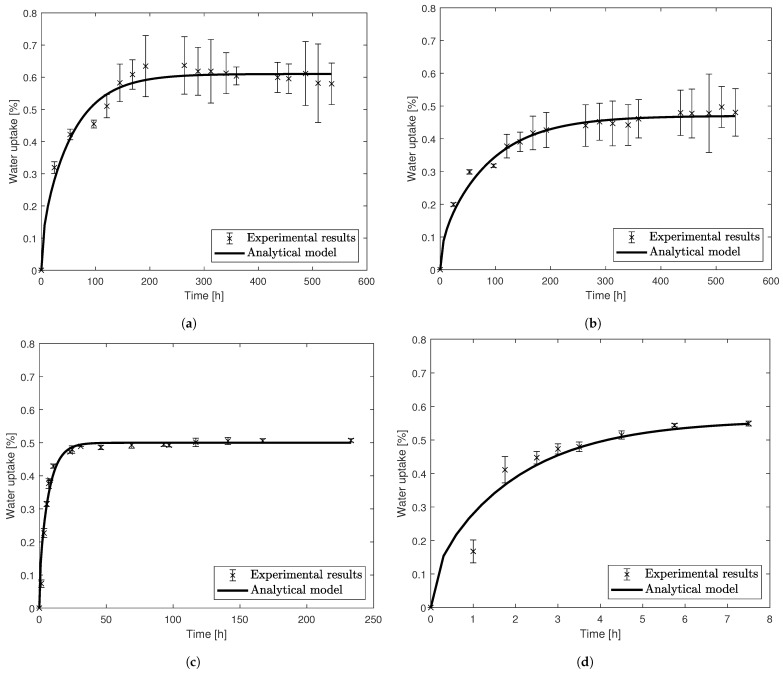
Experimental results and numerical details for the gravimetric tests. (**a**) PBT at 35 °C. (**b**) PBT-GF30 at 35 °C. (**c**) PBT-GF30 at 70 °C. (**d**) PBT-GF30 at 130 °C.

The experimental results and analytical curves obtained with the determined *D* and $M_{\infty }$ can be seen in Figure 4.

**Figure 5:**
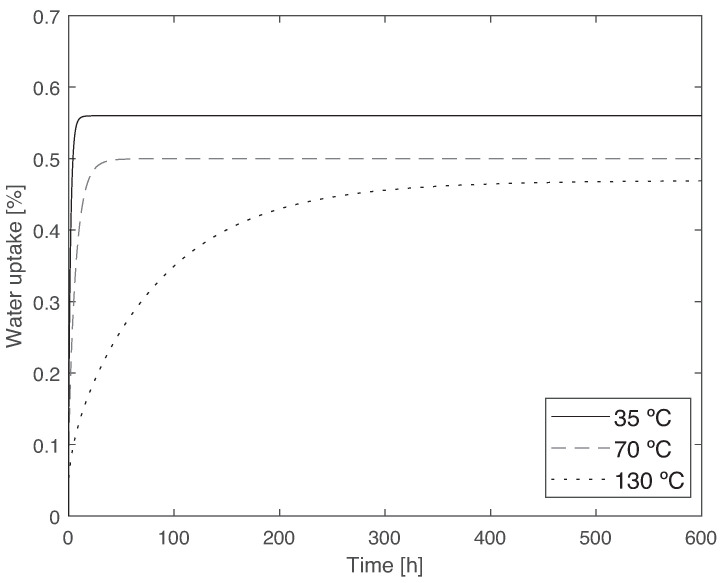
Comparison between the water uptake behavior of PBT-GF30 for 35, 70, and 130 °C, where it can be observed that the water uptake increases significantly with temperature, and the maximum water uptake also increases with temperature.

A comparison of the Fick’s law for the three different temperatures and for PBT-GF30 is given in Figure 5.

The results obtained show that fiber-free PBT absorbs more water than PBT with 30% of glass fibre. This may be due to the decrease in sites where the water molecules can form hydrogen bonds with the material, since those bonds are formed between the PBT matrix and water. Additionally, the material has lower porosity due to the portion of the material occupied by glass fibre, decreasing the water absorbed in the free volume of the polymeric chain of the PBT matrix. However, considering only this fiber/matrix ratio, the infinite water uptake of PBT-GF30 should be 70% of the infinite water uptake of fiber-free PBT, but the experimentally determined ratio is 77%, which implies that there are other factors playing a role in this, apart from the usual measurement accuracy consideration. These factors can be due, for instance, to capillarity effects along the fiber/matrix interface. Regarding the coefficient of diffusion, it is also higher for fiber-free PBT when compared with PBT-GF30. However, they have the same order of magnitude.

**Figure 6:**
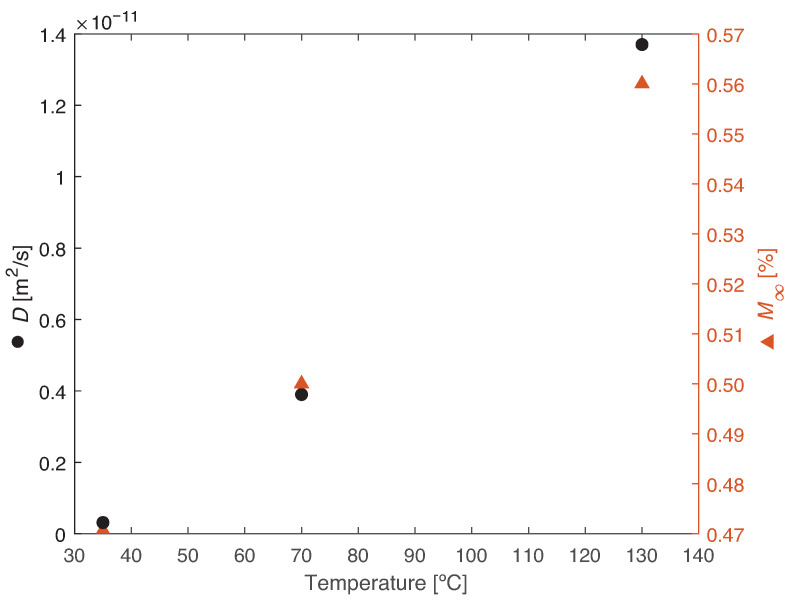
Diffusion parameters for PBT-GF30 as a function of temperature, showing an increase in the coefficient of diffusion and maximum water uptake.

PBT-GF30 absorbs a higher water content as temperature increases, the temperature-related absorption being smaller than that recorded between fiber-free PBT and PBT-GF30. Moreover, as temperature increases, the water absorption is significantly quicker, with the coefficient of diffusion being about two orders of magnitude higher. This phenomena occurs because at a higher temperature, there is an increase in energy and movement of the molecules, increasing the rate of diffusion. The infinite water uptake and coefficient of diffusion as a function of temperature can be seen in Figure 6.

The coefficient of diffusion can often be represented as a function of temperature by an Arrhenius relation, given by

(3) $$D=D_{0}\;\exp\left(\frac{-E_{A}}{RT}\right)$$

where $D_{0}$ is the permeability index, function of temperature, $E_{A}$ the activation energy for diffusion, *R* the universal gas constant, and *T* temperature. Rearranging Equation (3), it can be established that:

(4) $$\ln\left(D\right)=-\frac{E_{A}}{R}D_{0}\frac{1}{T}$$

**Figure 7:**
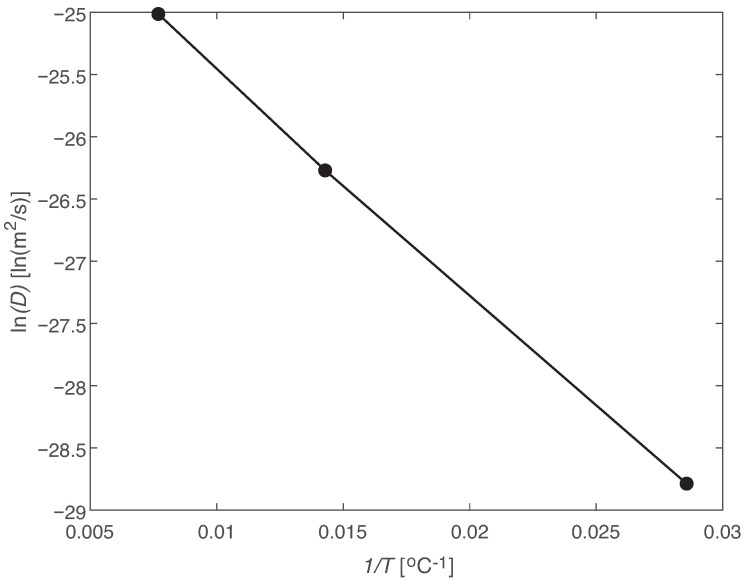
Arrhenius relation for the coefficient of diffusion.

Therefore, if *D* as a function of *T* follows an Arrhenius relation, ln(D) and 1/T will have a linear relation, Figure 7. From these results, $M_{\infty }$ as a function of temperature is given by a linear relation with a coefficient of determination, $R^{2}$, of 0.9985. The coefficient of determination of the linear relation between ln(D) and 1/T is 0.9996.

Among the results found in the literature, Bastioli et al. [48], studied the water uptake of fiber-free PBT at 37 °C and identified a coefficient of diffusion of 4.40 × $10^{-13}$ and an infinite mass of 0.65%, which is close to the results obtained for PBT at 35 °C. Ishak et al. [32], at 30 °C, identified a coefficient of diffusion of 1.90 × $10^{-13}$ for fiber-free PBT and 1.00 × $10^{-13}$ for PBT-GF30. Closer to the highest temperature studied in the present paper, at 100 °C, Ishak et al. [21] identified an infinite mass of 0.71% and a coefficient of diffusion of 4.05 × $10^{-11}$ for a content of fibers close to 30% (at about 28.5%).

### 3.4. Bulk Tensile Tests

#### 3.4.1. Validation of the Reduced-Scale “Dogbone” Specimens

**Figure 8:**
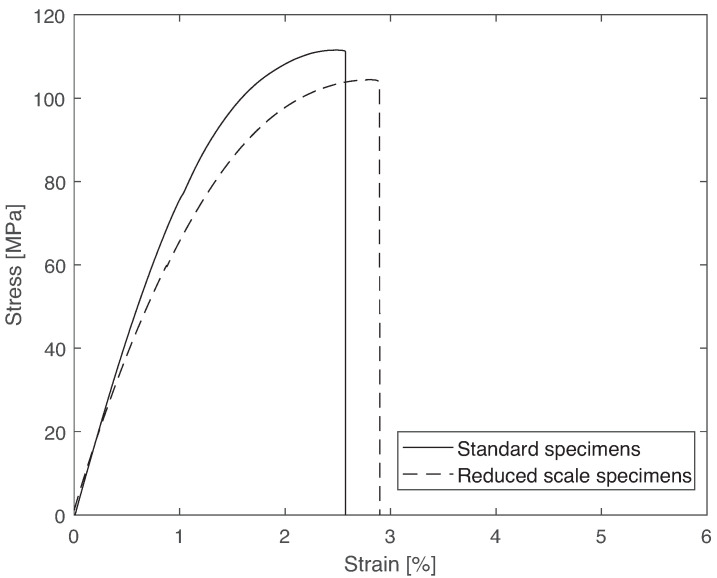
Validation of the reduced-scale “dogbone” specimens: representative stress vs. strain curves.

The representative stress vs. strain curves for standard and reduced-scale “dogbone” specimens can be observed in Figure 8, and the results are shown in Table 3.

It can be concluded that the reduced-scale specimens can be used to characterize the material as a function of temperature and immersion time. However, it is important to understand that the results obtained from standard specimens and reduced-scale specimens are not exactly the same. In previous studies [38] using the same specimens, strength increased compared to the standard “dogbone” specimens. Additionally, it was stated that the differences in Young’s modulus and strength were greater for more brittle materials. In this study, the difference in Young’s modulus and strength for the stronger and more ductile material were about 9 and 8%, respectively, and, for the more flexible and less ductile material, about 45 and 23%, respectively. In the present work, the error was of 5 and 6% for Young’s modulus and strength, respectively, which is lower that what was recorded for the previous work.

#### 3.4.2. Bulk Tensile Tests for Different Temperatures

**Figure 9:**
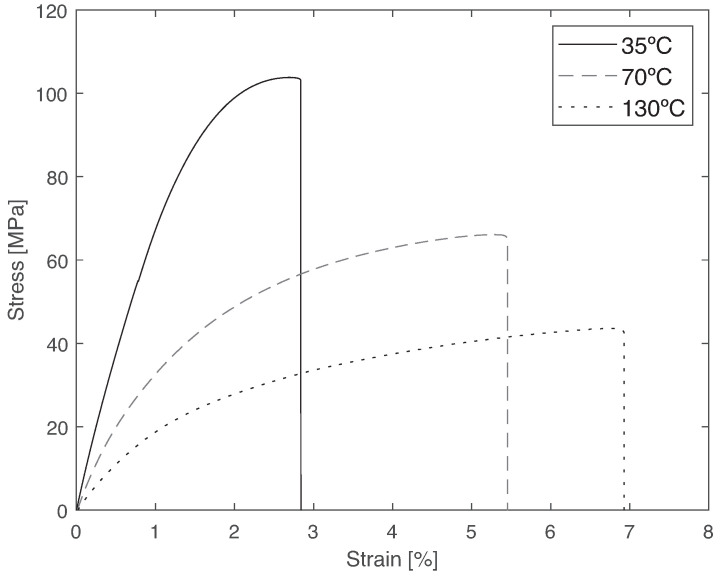
Bulk tensile tests at different temperatures: representative stress vs. strain curves, showing that stiffness and strength decrease with the testing temperature.

Representative stress–strain curves for the three temperatures analyzed are given in Figure 9, and the results are shown in Table 4.

**Figure 10:**
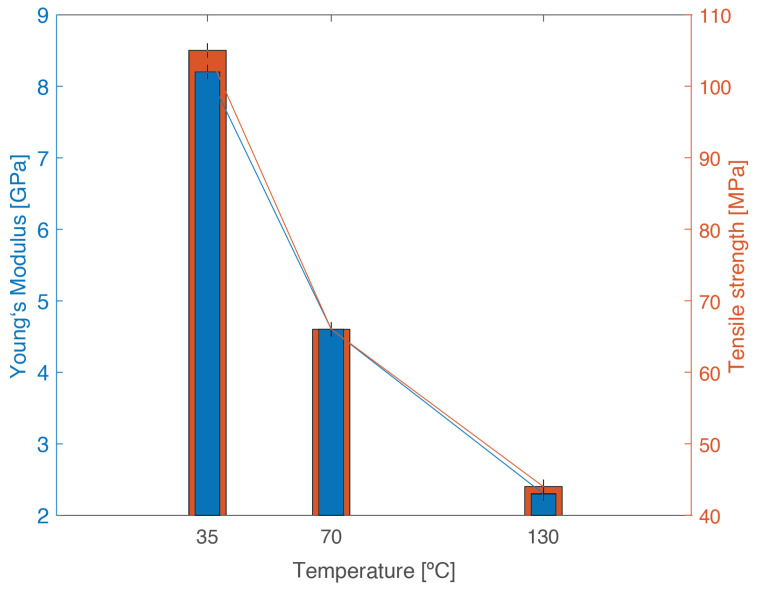
Tensile strength and stiffness of PBT-GF30 as a function of temperature, showing a decrease in both properties with increasing temperature.

From the experimental results, there is a clear decrease in both tensile strength and Young’s modulus with increasing temperature, (Figure 10). This decrease follows a similar trend for strength and Young’s modulus, being more severe between 35 and 70 °C, since $T_{g}$ is within these values. This behavior is typical of viscoelastic materials, whose properties depend on temperature. This happens because at a higher temperature, there is an increase in the mobility of the polymeric chain, which allows the accommodation of higher deformations; however, it contributes to a decrease in stiffness and strength.

#### 3.4.3. Bulk Tensile Tests after Aging at Different Temperatures

**Figure 11:**
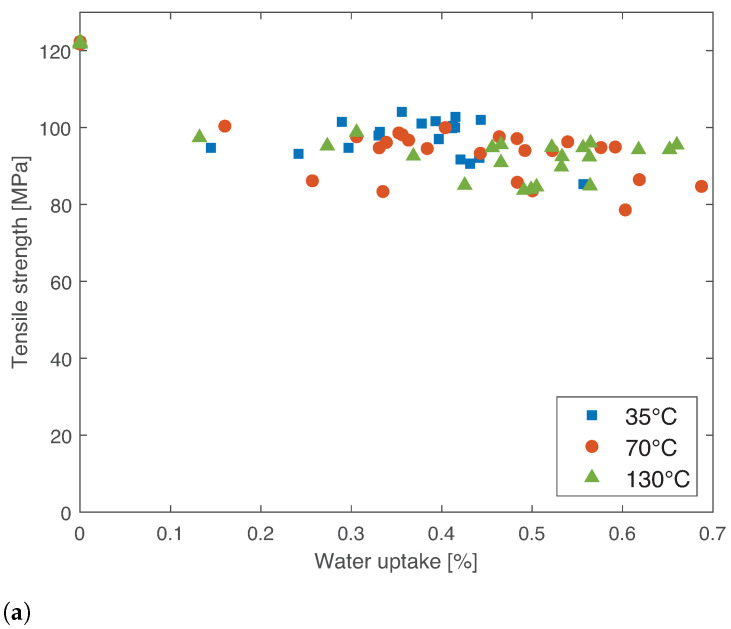

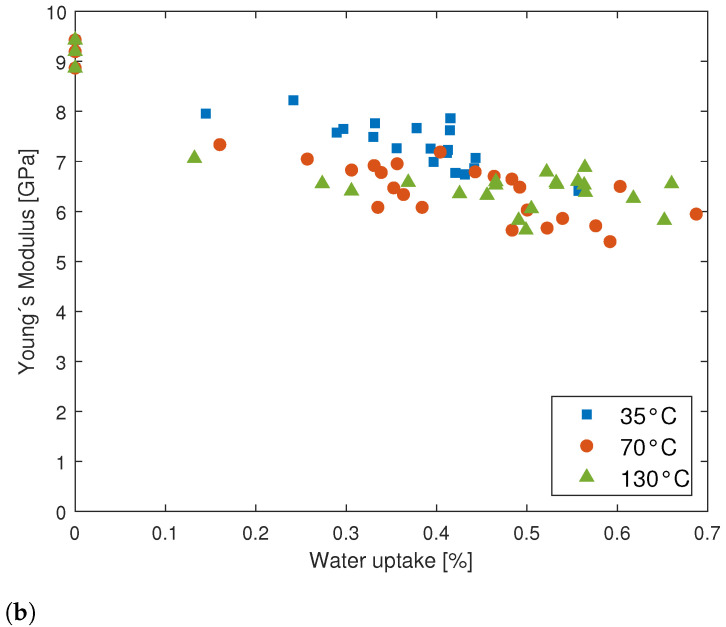
Tensile strength and stiffness of PBT-GF30, aged at 35, 70, and 130 °C as a function of water uptake. (**a**) Tensile strength. (**b**) Young’s modulus.

The results for tensile strength and Young’s modulus as a function of water uptake for 35, 70, and 130 °C are given in Figure 11.

It can be concluded that there is not a clear trend in any of the temperatures analyzed for the evolution of strength and Young’s modulus as a function of water uptake. However, it is clear that there is a decrease in both mechanical properties from the dry to the humid condition, since the material with humidity typically exhibits a relaxation on the polymeric chain. Additionally, the drop in mechanical properties is more significant for higher temperatures. Further explanation for these results can be found in Section 3.5 and Section 4.2. It is also important to highlight that in these tests, at RT, temperature does not play a role in the decrease in mechanical properties, only humidity, since when the specimen is tested, after weighing and correctly assembling on the universal testing machine, it is already at RT.

### 3.5. Fourier Transform Infrared Spectroscopy (FTIR)

**Figure 12:**
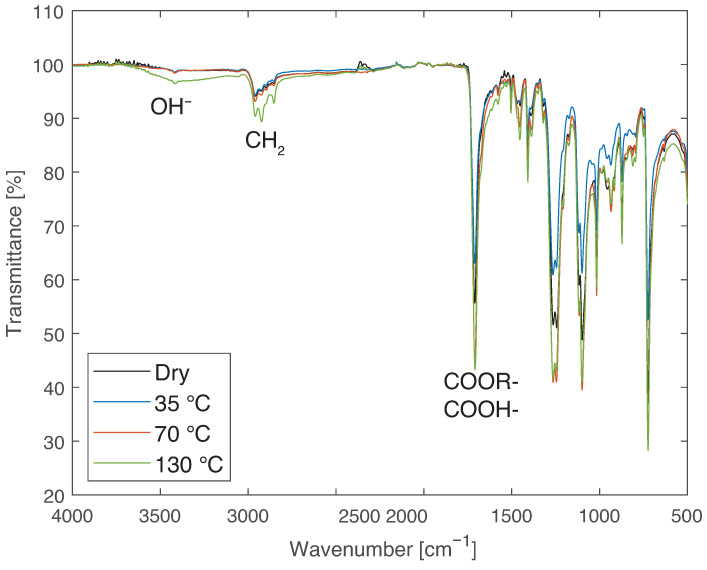
FTIR results of PBT-GF30 dry and after aging in water at 35, 70, and 130 °C, showing chemical changes found in the region related to OH${}^{-}$ for 130 °C.

The transmittance spectra obtained with the FTIR analysis can be seen in Figure 12.

From the FTIR analysis, it can be concluded that there is no significant change in the dry, 35, or 70 °C condition. However, for 130 °C, there is a difference in the range of from 3500 to 3000 cm${}^{-1}$, which is related to hydrogen bonds and may be caused by the establishment of chemical bonds with water or the hydrolysis of the material. This may be related to the larger decrease in mechanical properties for the higher temperatures.

**Figure 13:**
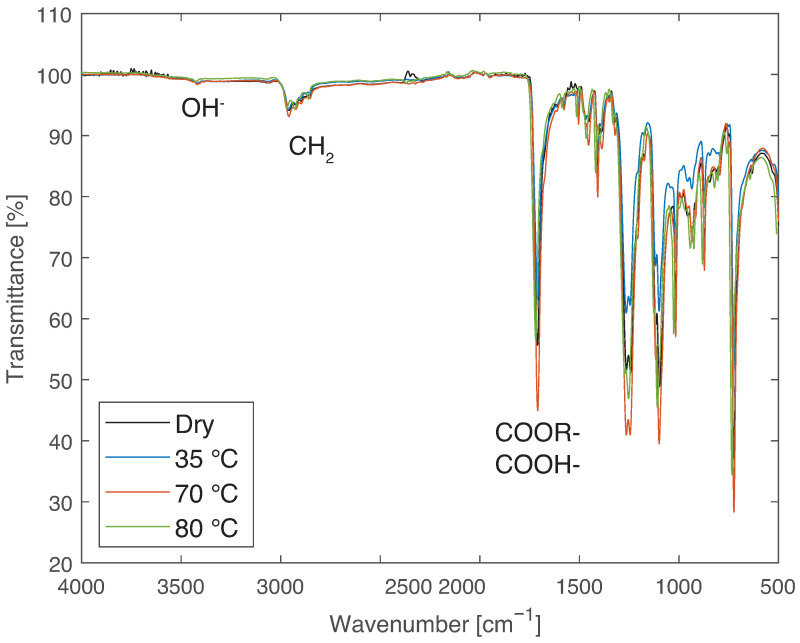
FTIR results of PBT-GF30 after aging in water at 35, 70, and 80 °C, showing no chemical changes found at these temperatures in the region related to OH${}^{-}$.

**Figure 14:**
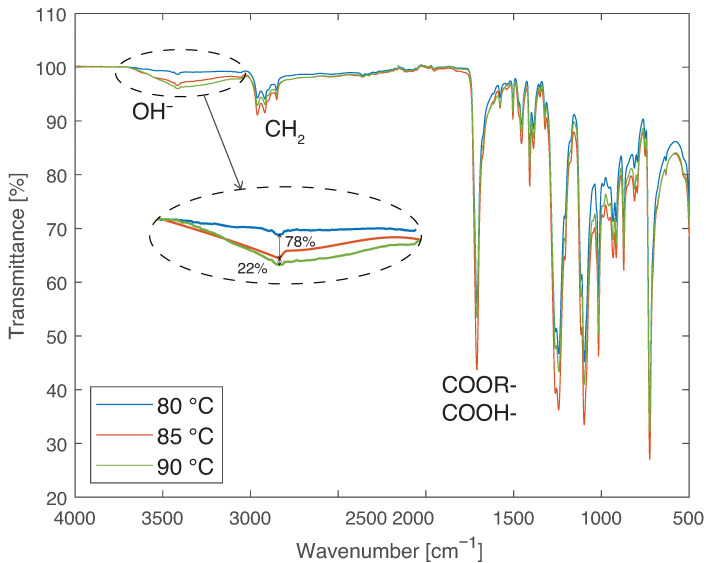
FTIR results of PBT-GF30 after aging in water at 80, 85, and 90 °C, showing chemical changes in the region related to OH${}^{-}$ appearing between 80 and 85 °C.

To complete the analysis and narrow the temperature range at which profile change happens, water immersion at other temperatures between 70 and 130 °C were analyzed. The first intermediate temperature analyzed was 90 °C. The analyzed range was gradually refined, leading to the conclusion that the change in FTIR profile of the material occurs between 80 and 85 °C. Figure 13 presents the results for 35, 70, and 80 °C, showing that no chemical bonds are formed until 80 °C is reached. Figure 14 exhibits the results obtained for those temperatures. From the curves for 80, 85, and 90 °C, further conclusions can be drawn. The peak in the curve for 85 °C, corresponding to the OH${}^{-}$ ions, in the zoomed area, is at 78% of the peak obtained for 90 °C. Since the curve for 90 °C is similar to that obtained at higher temperatures and the curve obtained at 80 °C is similar to the curve obtained at lower temperatures, it can be speculated that the exact transition temperature is closer to 80 °C than to 85 °C. However, to determine the exact values, further immersion temperatures need to be analyzed.

The chemical changes recorded can be either due to hydrolysis of the PBT or the creation of chemical bonds with water as bound water. In the first scenario, alcohol and acid functions should be formed, and analysing the FTIR spectra, the appearance of this functions can be seen at around 1750 cm${}^{-1}$. However, they are also present for low temperatures. Therefore, chemical bonds with water can be considered the most probable cause of the OH${}^{-}$ peak.

## 4. Numerical Simulation

### 4.1. Gravimetric Analysis

The main purpose of the developed numerical models of gravimetric studies was to create a methodology that is able to precisely reproduce experimental results. For this, first, two-dimensional approximation of the experimental tests was analyzed. This approximation is used to simplify the geometrically more accurate three-dimensional numerical model of the plate, significantly decreasing computation time. Afterwards, a second numerical model was developed to understand if the assumption that the water uptake is unidirectional is fair for these specimens in order to validate the analytical model. Finally, the experimental results were numerically reproduced.

#### 4.1.1. Evaluation of the Numerical Model Adopted

**Figure 15:**
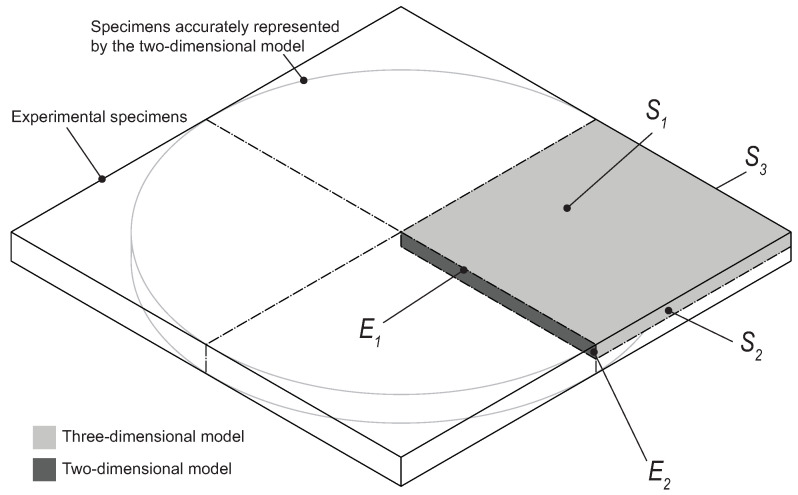
Schematic representation of the numerical model.

The specimens used in the experimental study are squares with a 60 mm side and 1 mm thickness. If one takes advantage of the symmetries in this structure, the section that must be simulated is that marked in dark grey in Figure 15. This model is referred to as three dimensional. In this model, water ingress must be considered through the surfaces $S_{1}$, $S_{2}$, and $S_{3}$.

However, this three-dimensional model is computationally heavy, both when running the simulation and the post-processing operations. Therefore, a simplified two-dimensional model was developed in an effort to obtain similar results but with reduced computational time. This two-dimensional model only considers the slice represented in dark grey in Figure 15. Water uptake in this specimen is considered to occur through edges $E_{1}$ and $E_{2}$.

This slice represents an oversimplified model of the experimental tests, which would only accurately represent circular specimens, as presented in Figure 15. Although the corners present in the experimental specimen are neglected, if the results are not significantly affected by this simplification, it has the potential to lead to an important reduction of computation time. Therefore, the two-dimensional and three-dimensional models were run, and their results compared.

**Figure 16:**
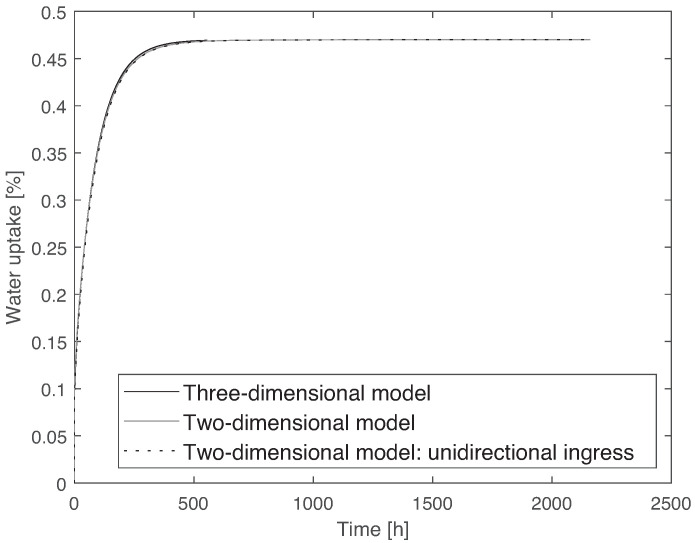
Comparison between the three-dimensional, two-dimensional, and two-dimensional with unidirectional ingress models, using data from PBT-GF30 at 35 °C, showing that a two-dimensional model can be used.

To simulate water diffusion, a heat transfer analogy was used, where the coefficient of diffusion can be compared to the thermal diffusivity. It is also considered that once the material is immersed in water, the first layer of nodes instantaneously saturates, which means a boundary condition is applied with the value of water uptake at saturation. For the two-dimensional model, a uniform mesh of $5\cdot 10^{-5}$ m was used, and for the three-dimensional model, a mesh size of $2\cdot 10^{-4}$ m was used. Due to the large computational time of the three-dimensional model, it was only run until the saturated level was reached. The elements used are heat transfer elements: for the two-dimensional model, a 20-node quadratic heat transfer brick (DC3D20), and for the three-dimensional model, a 4-node linear heat transfer quadrilateral (DC2C4). The results of water uptake as a function of time for the two models can be seen in Figure 16. From these results, it can be concluded that a two-dimensional approximation can be used. The plates used in this work have a thickness significantly smaller than the other dimensions of the specimen, with a 1:60 ratio. Therefore, it seems fair to assume that water is absorbed mainly through the thickness. However, to ensure this is an appropriate approximation, a numerical simulation was conducted. With this purpose, the two-dimensional model represented in Figure 15 was compared to a model similar to it but taking into account that the side surfaces were isolated, i.e., water uptake only occurs through $E_{1}$. This model is referred to as two-dimensional model with unidirectional ingress. The two models were run, and a comparison between them can also be observed in Figure 16. From this numerical study, it can be concluded that a unidirectional water ingress occurred in the experimental tests.

From Figure 16, it is clear that there is a slight difference between the two-dimensional and the three-dimensional models, which is due to the aforementioned simplification of the corners. However, it is clear that the two-dimensional model is quite capable of accurately describing the water uptake behavior and, due to the lower computation time, this was the model adopted for the remaining work.

#### 4.1.2. Simulation of the Experimental Results

**Figure 17:**
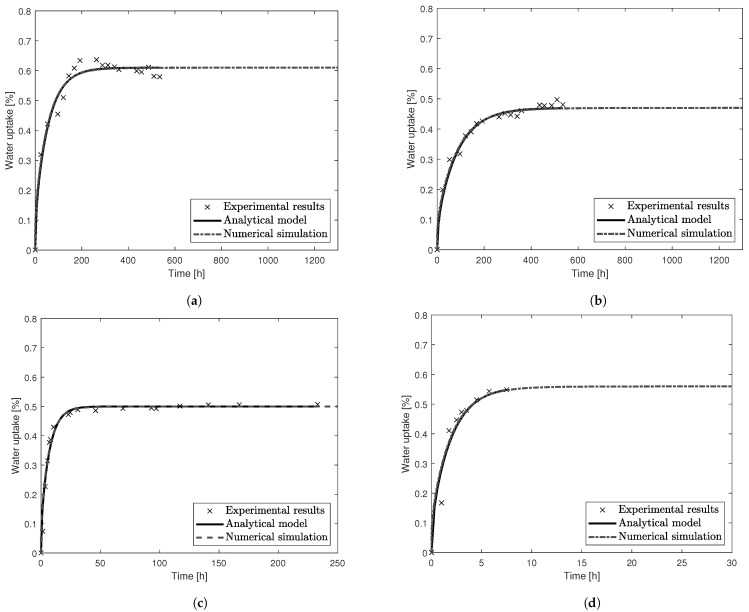
Numerical simulation of water uptake for the gravimetric tests compared with the analytical model and experimental results, showing that the simulation fits the analytical model. (**a**) PBT at 35 °C. (**b**) PBT-GF30 at 35 °C. (**c**) PBT-GF30 at 70 °C. (**d**) PBT-GF30 at 130 °C.

A numerical simulation was conducted to mimic the water absorption behavior of the PBT and PBT-GF30 plates, using a two-dimensional model considering water uptake through the exposed surfaces, similar to that previously described as a two-dimensional model. For each simulation, the coefficient of diffusion and maximum water uptake were set to the values presented in Table 2. A comparison between the analytical model established to describe the water absorption behavior and the results of the numerical simulation is shown in Figure 17.

It can be concluded that the developed numerical simulation exhibits a very good correlation with the obtained experimental results.

#### 4.1.3. Minimum Plate Side Length–Thickness Ratio for the Water Flow to Be One Dimensional

In this study, one of the concerns identified regarding the experimental procedures was the large amount of material required to manufacture the plates for the gravimetric study. Since the plates were obtained from injection-molded PBT and PBT-GF30, it would be beneficial to use smaller plates so that the defects introduced to the surface by injection molding could be avoided, thereby avoiding sandpaper treatment. Additionally, in multi-material structures, or in parts where there is an aim to characterize a smaller portion of the material, smaller plates would also be beneficial. However, the water flow in these smaller plates must remain unidirectional and, from the numerical point of view, it is beneficial to maintain the valid two-dimensional approximation.

**Figure 18:**
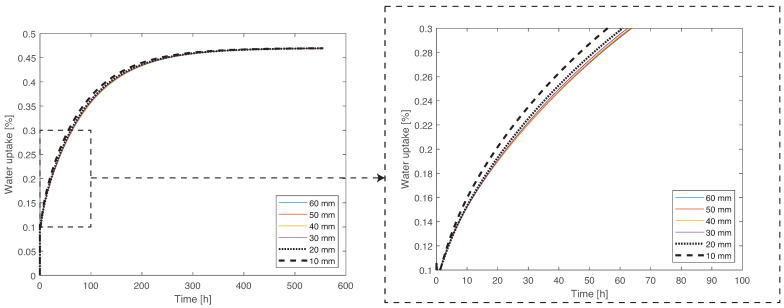
Numerical simulation of the water uptake of specimens with different sizes at 35 °C, showing that the results are in agreement for the 60 and 30 mm side lengths.

Therefore, as a first step, a three-dimensional model, described above, was compared for different side lengths, which differ by 10 mm (60, 50, 30, 20, and 10 mm), always maintaining a thickness of 1 mm (Figure 18). The mesh size of these models was changed for each plate, in a manner proportional to the side length, to maintain the number of elements.

**Figure 19:**
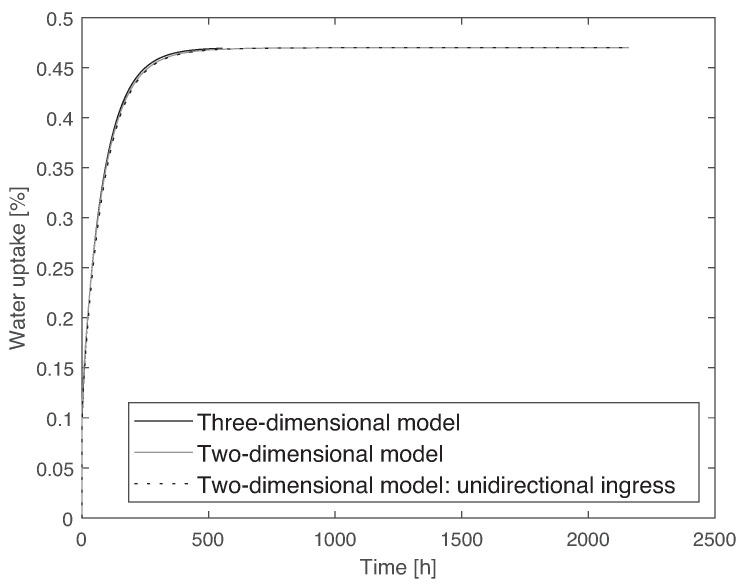
Comparison between the three-dimensional, two-dimensional, and two-dimensional with unidirectional ingress models for a plate with a 30 mm side length, using data from PBT-GF30 at 35 °C. As can be observed, the two-dimensional model remains valid.

The side length of 10 mm was discarded from consideration at an early stage, since the difference between the 60 mm and 10 mm plates is significant, with a maximum error of 6%. The size of 20 mm presents lower discrepancy in the results, with a maximum error of 2%. However, a plate with a 30 mm of side was considered to present the best compromise between the fidelity of the water uptake process, with a maximum error of 1%, and the amount of material necessary for its manufacture. Afterwards, the model was used to determine if the two-dimensional approximation used previously remained valid and if the water flow remained unidirectional, which is the case, as can be observed in Figure 19.

At this point, it is understood that, from the numerical point of view, a plate with a 30 mm side length would be suitable to characterize the water uptake behavior of PBT-GF30. Although the coefficient of diffusion and water uptake at saturation used to conduct this simulation are obtained with water immersion at 35 °C, the same correlation would be found for 70 and 130 °C.

**Figure 20:**
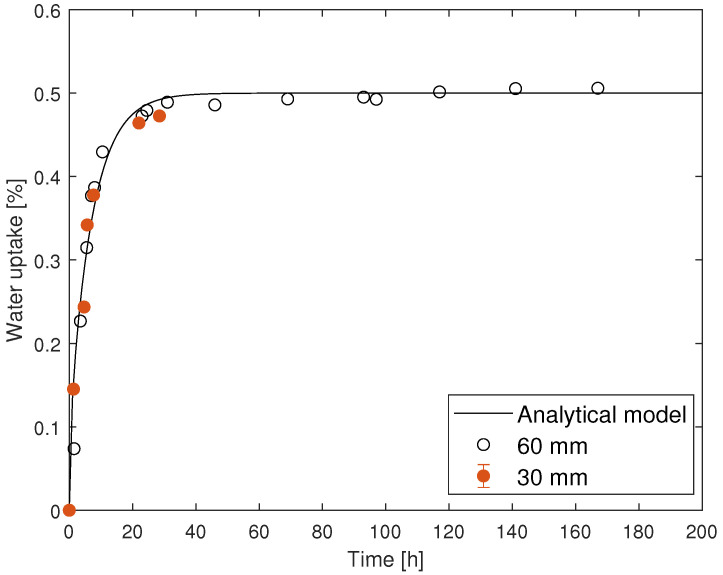
Experimental validation of a plate with a size of 30 mm, showing that this plate size is sufficient for water uptake behavior characterization.

Experimental validation was performed at 70 °C to avoid the long immersion time required to saturate the material at 35 °C and to avoid chemical changes that would occur at 130 °C. Figure 20 shows that the water uptake behavior obtained with plates with a 30 mm side length have similar experimental results to those with a 60 mm side length.

From these results, it is concluded that plates with a side length of 30 mm and a 1 mm thickness can be used to perform gravimetric studies.

### 4.2. Simulation of the Bulk Tensile Tests

**Figure 21:**
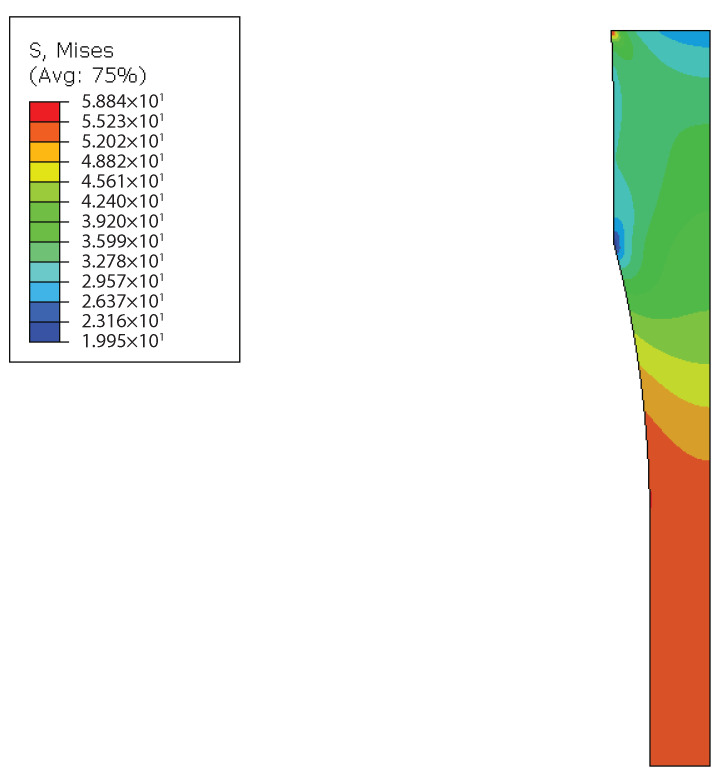
Numerical simulation of the bulk tensile tests using reduced-scale specimens, showing higher stress near the edge of the specimen.

The bulk tensile tests at room temperature using the reduced-scale specimens were simulated in Abaqus using one-eighth of the specimen, taking full advantage of symmetry. One of the edges was pinned, and displacement was applied to the opposite side. In this model, a uniform mesh of 0.2 mm was used, and both the elastic and plastic properties of the material were considered. The mesh and stress in the time increment before plasticization starts can be observed in Figure 21. The elements used are 3D stress, an 8-node linear brick, and reduced integration (C3D8R). This simulation was conducted from the properties obtained in the as-supplied state when tested at room temperature as a given condition to evaluate stress distribution for bulk tensile tests.

It can be observed that there is a stress gradient from the edges to the middle of the specimen, with stress being higher at the edges. Therefore, it can be assumed that the specimen starts to fail at those edges, damaged by water uptake.

**Figure 22:**
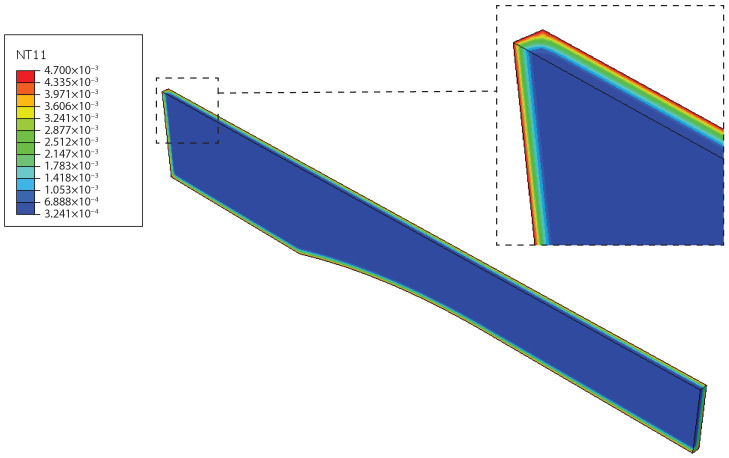
Numerical simulation of the water uptake, NT11, for reduced-scale specimens at 35 °C at the end of 21.2 h, showing that at this stage, only the outer layers are saturated.

Reduced-scale specimens were also simulated in Abaqus. Taking advantage of symmetry, only one-eighth of the specimen was represented. Additionally, the same boundary conditions described above for the gravimetric analysis simulation were used. In Figure 22, water distribution was achieved after one day and a half of water immersion at 35 °C. The global water uptake level at this point in the specimen was 0.26% according to the numerical simulation.

From these two simulations, it can be observed that as the water ingresses the material, the first region to saturate is the outer layer, which is also the region where the stress for a given time increment of the bulk tensile test is higher. The core of the material, as shown in Figure 22, has a significantly smaller water content. However, the specimen starts to fail at the saturated edge, which is almost immediately saturated after water immersion. Consequently, all humid states depend on the same condition to initiate failure, which is failure of the saturated edge. This translates into similar strength of all humid conditions.

The authors that also studied the effect of water uptake on the mechanical properties of PBT and its short fiber composites conducted slightly different experiments, which may have led to different results. The differences are mainly regarding immersion time and specimen thickness.

Bergeret et al. [34], when analyzing PBT with 15% short glass fiber using “dogbone” immersed in water at 120 °C, concluded that for short immersion times, the strength of the material decreased, reaching a stable *plateau*. After a longer immersion time, strength was found to undergo a second drop. In the first decrease, for short immersion times, strength decreased to just above 80 MPa, which is similar to the results obtained in this work.

Ishak et al. [31], as discussed above in Section 1, reported a decrease to 21% in the initial strength and to 36% for PBT-GF30 (when compared to the unaged state) after water immersion at 90 °C. In this study, specimens were tested at 20 °C when saturated. However, the immersion time may correspond to the second drop in strength. Additionally, the decrease in strength described by this work for 30 °C is compatible to that identified in the present work for 35 °C.

Rosato et al. [23] and Gardner et al. [35], as mentioned above in Section 1, also noted that the strength of PBT exposed to environments of 100% of relative humidity can be significantly decreased after very long exposure times. After three to four years, the tensile strength was reduced to about 50%.

The other variable that may influence the results is temperature. Although it is common for tests to be performed at room temperature after water immersion at a higher temperature, the specimens used in the present study have a smaller thickness to decrease immersion time, which may lead to a quicker decrease in temperature between the time the specimen is removed from the climatic chamber and the end of the test. Therefore, temperature plays a less significant role, leading to a higher strength and Young’s modulus.

## 5. Conclusions

The main conclusions drawn from this work are that the diffusion for all conditions tested follows a Fickian law. Fiber-free PBT absorbs more water than PBT-GF30, which may be not only attributed to the portion of the hydrophilic matrix and hydrophobic fibers but also to the capillarity between the fiber and the matrix. Regarding the rate of water ingress, it is slightly higher for material without fiber inclusions. However, the main influence on this rate occurs due to temperature following an Arrhenius relation. The effect of temperature on the mechanical properties of PBT-GF30 is more significant than the effect of humidity.

For humid conditions at a given temperature, the mechanical properties obtained through tensile testing specimen do not change as a function of water uptake due to the fact that the outer layer of the specimen material is saturated in every case, corresponding to the region of maximum stress during the test.

Regarding chemical degradation of the material, after water immersion at a temperature between 80 and 85 °C, there is the establishment of chemical connections between water and PBT or hydrolysis of PBT (degradation of the polymer chain), which produces alcohol and carboxylic groups, both exhibiting OH groups.

Additionally, with this work, it was possible to conclude that plates with a 30 mm side length can be used to trace the water absorption profiles of a material with similar results to those obtained following the standard larger plates. This is valid for 1 mm-thick plates.

## Figures and Tables

**Table 1:** Glass transition temperature measurement results.

Material	$T_{g}$ (°C)
PBT-GF30	49 ± 4
PBT	54 ± 6

**Table 2:** Water uptake properties of the materials analyzed.

Material	*T* (°C)	*D* (m${}^{2}$/s)	$M_{\infty }$ (%)
PBT	35	$4.80\times 10^{-13}$	0.61
PBT-GF30	35	$3.15\times 10^{-13}$	0.47
PBT-GF30	70	$3.90\times 10^{-12}$	0.50
PBT-GF30	130	$1.37\times 10^{-11}$	0.56

**Table 3:** Mechanical properties of standard and reduced-scale “dogbone” specimens at room temperature, showing that the results for stiffness and tensile strength have a small error.

Property	Standard Specimens	Reduced-Scale Specimens	Error
Young’s modulus (GPa)	8.6 ± 0.1	8.2 ± 0.1	4.6%
Tensile strength (MPa)	112 ± 1	105 ± 1	6.3%

**Table 4:** Bulk tensile tests at different temperatures and stiffness tensile strengths, showing the stiffness and strength decrease with testing temperature.

Property	35 °C	70 °C	130 °C
Young’s modulus (GPa)	8.2 ± 0.1	4.6 ± 0.1	2.3 ± 0.1
Tensile strength (MPa)	105 ± 1	66 ± 1	44 ± 1

## Data Availability

No new data were created or analyzed in this study. Data sharing is not applicable to this article.
